# LiquidGAN for Handwriting-Based Detection and Severity Classification of Extrapyramidal Symptoms

**DOI:** 10.3390/s26123890

**Published:** 2026-06-18

**Authors:** Erandhi M. Liyanage, Chun-Hung Lee, Wen-Yen Chang, Andrew An-Zhe Lee, Guan-Hsiung Liaw, Wu-Chuan Yang, Yu-Hsin Liu, Kun-Chan Lan, Sai Ho Ling

**Affiliations:** 1 School of Electrical and Data Engineering, University of Technology Sydney, Sydney, NSW 2007, Australia; 2 Jianan Psychiatric Center Ministry of Health and Welfare, Tainan City 701, Taiwan; yuhsinliu87@gmail.com; 3 Taoyuan Psychiatric Center, Ministry of Health and Welfare, Taoyuan City 330, Taiwan; anzhe0327@gmail.com; 4 Department of Computer Science and Information Engineering, National Cheng Kung University, Tainan City 701, Taiwan; d11452002@gapps.ntust.edu.tw (W.-Y.C.); 9602016@gs.ncku.edu.tw (K.-C.L.); 5 Department of Information Engineering, I-Shou University, Kaohsiung City 800, Taiwan; ghliaw@isu.edu.tw (G.-H.L.); wcyang@isu.edu.tw (W.-C.Y.); 6 Florence Nightingale School of Nursing & Midwifery, King’s College London, London WC2R 2LS, UK; liushin1112@gmail.com

**Keywords:** extrapyramidal symptoms, handwriting analysis, liquid neural networks, generative adversarial networks, deep learning, antipsychotic medication monitoring

## Abstract

Extrapyramidal symptoms (EPS) are motor side effects commonly induced by antipsychotic medications and can lead to measurable changes in handwriting patterns. These symptoms affect both the spatial and temporal characteristics of writing, including stroke thickness, direction and the rate of directional change. To model these complex variations, we propose a novel Liquid Generative Adversarial Network (LiquidGAN), which combines the adaptive dynamics of liquid neural networks with the data generation capability of GANs. Handwriting data were collected from 94 patients with confirmed EPS and 30 healthy controls using Archimedean spiral patterns drawn with both hands. A total of 211 images were processed for both binary and multiclass classification using a pretrained ResNet50 model. The pretrained ResNet50 achieved 92% accuracy and 97% precision in the binary classification task; however, its performance dropped significantly to 57% accuracy in multiclass classification, indicating limited capability in capturing fine-grained EPS severity variations. In contrast, the proposed LiquidGAN demonstrated excellent performance in the binary classification task, achieving 97% accuracy and 98% precision. More importantly, LiquidGAN substantially outperformed the baseline in the more challenging multiclass setting, achieving 70% accuracy and precision across four classes (mild, moderate, severe, and control). This shows that the diverse dataset from the liquidGAN significantly improves the HOG-ANN classification and effectively captures complex and subtle handwriting variations associated with different EPS severity levels that conventional models such as ResNet50 fail to distinguish. In addition, LiquidGAN generated diverse and realistic synthetic handwriting samples, yielding improved Fréchet Inception Distance (FID), precision, and recall compared with style GAN. These findings demonstrate that handwriting biomarkers, when analyzed through dynamic generative learning, offer an effective and non-invasive approach for monitoring extrapyramidal side effects in clinical settings.

## 1. Introduction

Extrapyramidal symptoms (EPS) are adverse effects induced by the intake of antipsychotic (AP) medications. These symptoms arise as a result of blocking Dopamine 2 receptor (D2R) at the nigrostriatal dopamine pathway [1]. The severity of EPS depends on the type of antipsychotic medication administered, and for this reason they are also referred to as drug-induced movement disorders. EPS are commonly characterized by dystonia, tardive dyskinesia, akathisia, and parkinsonism [2]. Parkinsonism motor symptoms are often represented by cogwheel rigidity, dystonia, and bradykinesia and posturalinstability [3,4]. Such motor dysfunctions can manifest not only in physical movements but also through observable alterations in handwriting and voice patterns.

Psychiatrists prescribing antipsychotic medications cannot always foresee the extent of EPS severity in advance. Typically, treatment is prescribed and the severity of symptoms is monitored using either subjective or objective physical examination scales. The Glasgow Antipsychotic Side Effect Scale (GASS) is a subjective, self-reported scale ranging from 0 to 63, where 0–21 indicates absent or mild side effects and 43–63 indicates severe side effects [5]. The Drug-Induced Extrapyramidal Symptom Scale (DIEPSS) is an objective measure evaluated by a trained psychiatrist, with scores ranging from 0 (normal) to 4 (severe) across 8 individual symptoms [6]. Both scales assess bradykinesia, muscle rigidity, tremors, restlessness, and postural instability [5,6]. Importantly, these parkinsonism-related symptoms explicitly affect handwriting patterns.

The deterioration of handwriting associated with motor impairment has been long recognized, dating back to James Parkinson’s 19th century observations of handwriting changes in patients with Parkinson’s disease. Handwriting modifications in patients with schizophrenia spectrum and bipolar disorders include alterations in acceleration, velocity, and writing pressure [7]. Tremor types can be classified into essential, dystonic, functional, and parkinsonian based on distinct stroke patterns [8,9]. Observing handwriting allows clinicians to identify abnormalities such as bradykinesia, tremor, postural instability, and distractibility [8]. The Archimedean Spiral Rate (ASR) has been used to measure tremor severity, with higher ASR scores observed in males and increasing with age [10]. Women generally demonstrate lower tremor severity in their dominant hand [10]. Archimenedians spiral test has been shown to be highly sensitive for essential tremor. It is standardized compared to free writing, meanders, loops and straight line which are commonly used as a handwriting test for parkinsonism [8,11]. Both Parkinson’s disease and aging influence fine motor skills, such as handwriting [9]. Several studies have therefore incorporated deep learning models to identify and classify Parkinsonism from handwriting data.

CNN-based and pretrained CNN models were selected as baseline methods because they are among the most widely adopted architectures for handwriting analysis in Parkinsonism. Previous studies have demonstrated strong performance using these approaches. For example, a pretrained VGG model achieved 96.67% accuracy for Parkinson’s disease detection using spiral and wave drawings [12], while a hybrid CNN model reported accuracies of 87.93% and 87.24% for spiral and wave drawing tasks, respectively [13]. A recent study showed that a CNN extracting spatial features achieved 100% accuracy in detecting PD, though only a small dataset was utilized, the classification was only binary [14]. Furthermore, Cure-Net [15] reported an accuracy of 92.5% using meander and spiral drawing images.

While numerous studies have analyzed handwriting in Parkinson’s disease focusing on stroke patterns, tremor severity, and predictive models of disease progression research into drug-induced parkinsonism (DIP) remains limited. One study specifically investigated tremors induced by antipsychotic medications using a random forest model, reporting 100% accuracy, but only for a binary classification of tremor versus no tremor [16]. Another study demonstrated increased stroke length, pressure, and disfluency, along with reduced velocity and acceleration, in patients taking antipsychotics [7]. However, no predictive model was developed from these findings. A single predictive model examining the tremor aspects of EPS was developed by Wysokiński and Zwierzchowska-Kieszek [16]. However, this predictive model focused only on tremor in EPS and not on the overall severity of the condition.

A major challenge in developing deep learning models for medical data is the finite quantity of available training samples. Since the robustness of deep learning models depends on both the quality and quantity of data, generative models such as Generative Adversarial Networks (GANs) have been proposed to synthesize additional data samples. GANs consist of two networks: a Generator (*G*), which attempts to mimic the true data distribution, and a Discriminator (*D*), which learns to distinguish between real and generated data [17]. More recent models, such as StyleGAN, address some of the limitations of the original GAN architecture, including training instability, mode collapse, entangled latent space, lack of control, and visual artifacts [18]. The performance of generative models is commonly evaluated using metrics such as Inception Score (IS), Fréchet Inception Distance (FID), Precision, Recall, Kernel Inception Distance (KID), and Mode Score [18,19,20]. However, conventional GANs utilize static neural networks which require high computational power and exhibit limited adaptability [17]. Liquid neural networks, which model neuron dynamics using differential equations, offer dynamic learning with real time feedback [21]. ODE physics-based equations are used for detection of mechanical tremor [14]. Utilizing these liquid learning capabilities in the generative model would improve its generative capacity.

In this study, we aim to address the limited research in handwriting biomarkers for the early detection of EPS. By developing a predictive model to determine the EPS severity using static handwriting Archimedean spiral patterns, we overcome the challenge of small, imbalanced datasets by proposing a differential equation based Liquid GAN framework that dynamically learns temporal and spatial handwriting features. By synthesizing diverse, high-quality handwriting samples, our approach enhances classification of EPS severity into mild, moderate, severe, and control groups. This study provides an objective, non-invasive method that could complement existing clinical scales for monitoring antipsychotic side effects.

### Contributions

Research on handwriting pattern alterations associated with EPS remains limited. This study addresses this gap by developing and validating a dynamic LiquidGAN framework for early detection and severity assessment of EPS. The major contributions of this work are as follows:

1. Comprehensive handwriting analysis:We conducted a detailed quantitative analysis of handwriting features, demonstrating that horizontal skewness, stroke thickness, dominant direction, and directional change rate vary significantly with EPS severity across medicated and non-medicated groups. However, the feature changes are not proportional to EPS severity. These findings provide strong empirical evidence for handwriting as a reliable biomarker.
2. Novel LiquidGAN framework with generative capability: We propose a generative adversarial network integrated with liquid neural dynamics that produces high-quality and diverse handwriting samples (Precision: 85%, Recall: 81%, IS: 4.5, FID: 165.33). The framework demonstrates enhanced adaptability and representation capability, enabling effective modeling of complex handwriting variations.
3. High performance binary classification using LiquidGAN: The proposed LiquidGAN framework achieves excellent performance in binary classification, attaining 97% accuracy and 98% precision, demonstrating its robustness in distinguishing EPS from healthy controls.
4. Significant improvement in multiclass EPS severity classification:The proposed framework achieves 70% accuracy and precision in classifying EPS severity into four categories (mild, moderate, severe, and control), substantially outperforming the baseline ResNet50 model, which achieved 57% accuracy. This highlights the core contribution of this work: LiquidGAN, with its dynamic learning capability, significantly improves multiclass classification performance.

These contributions collectively advance objective and non-invasive assessment methods for antipsychotic-induced motor side effects, bridging the gap between clinical diagnosis and digital biomarker discovery. This research, to the best of our knowledge, is the first handwriting-based EPS classification model.

## 2. Materials and Methods

**Figure 1:**
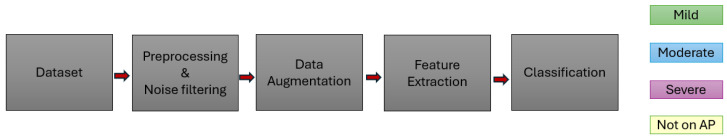
The handwriting data flow from collection, processing to the final classification.

The overall workflow of the proposed system is illustrated in Figure 1. Handwriting data were collected, preprocessed, and used for feature extraction, data augmentation, and classification of extrapyramidal symptom (EPS) severity.

### 2.1. Dataset

The dataset used in this study was provided by a psychiatric center in Taiwan. All patients recruited exhibited Parkinsonism symptoms and were receiving antipsychotic (AP) medication at the time of recruitment. A detailed medication history was collected for each patient. A balanced distribution of male and female participants aged between 21 and 60 years was included. The dosage, duration, and type of medication were not controlled or kept constant. In addition, 30 participants not receiving antipsychotic medication were recruited as a control group.

All enrolled participants were asked to complete the Archimedean Spiral Test. Each participant completed two spirals, one dotted and one continuous, using both their dominant and non-dominant hands. The drawings were recorded on paper then converted to PNG format. A total of four spiral drawings per participant were collected, resulting in two PNG images per participant. To reduce variability associated with handwriting behavior, all participants completed a standardized Archimedean spiral drawing task under controlled experimental conditions. In addition, both left- and right-hand spiral patterns were collected to minimize potential bias related to hand dominance. These measures helped improve consistency across participants and testing sessions.

A total of 211 PNG images were collected and divided into four classes: EPS Mild (53), EPS Moderate (66), EPS Severe (32), and Not on Antipsychotic (60).

#### Classification into EPS Severity

The severity of EPS was assessed by a specialist doctor using both objective and subjective scales. A trained clinician conducted the Drug-Induced Extrapyramidal Symptom Scale (DIEPSS), which assesses eight EPS induced symptoms: gait, rigidity, dyskinesia, dystonia, akathisia, sialorrhea, and bradykinesia, ranked on a scale from 0 (normal) to 4 (severe), along with a global average score [6]. The Glasgow Antipsychotic Symptom Scale (GASS) was self-appraised by the patients. This included 16 questions related to the side effects of AP, from dry mouth to bradykinesia. Patients were asked to give a score between 0 and 3 depending on the frequency of occurrence, with 0 being never and 3 being most frequently [5].

The final labeling of mild, moderate, or severe was checked along with the scores of the DIEPSS and GASS scales before classifying patients into these classes. The participants not receiving any AP medication were labeled as the not on antipsychotic group. The individual files were labeled with the participant ID and sample number, such as 00030_02.

The Archimedean Spiral Rating included the two types of spirals that the enrolled participants had to complete using both their dominant and non-dominant hands. As each patient has either right or left as their dominant hand, either hand shows different types of features, such as tremor. The continuous and the non-continuous spirals were used to test various aspects of the symptoms.

### 2.2. Preprocessing and Noise Filtering

The raw spiral images were processed with median and bilateral filters, along with background subtraction, deskewing, and normalization.

The median filter replaces each pixel with the median of its 5×5 neighborhood, removing impulse salt-and-pepper noise [22] which averages the pixel values.

The bilateral filter was employed to denoise the image while preserving the edges by combining spatial and intensity-based filtering. A filter diameter, spatial sigma, and intensity sigma of 9, 75, 75 were utilized. This ensures that fine structures such as text edges are preserved.

Background subtraction was then applied to eliminate shadows, dynamic backgrounds, and camera jitter [23] using Gaussian weighting with size 11 to eliminate uneven illumination.

Finally, deskewing and normalization were applied to straighten the skewness of the scanned images. The image size was standardized to 64×64 pixels and divided by 255 to normalize pixel intensities.

### 2.3. Data Augmentation

A power analysis based on a one-sample proportion test was performed using chance-level accuracy of 25%, an expected accuracy of 90%, α=0.05, and statistical power of 0.80. Although the theoretical minimum sample size was small due to the large assumed effect size, the model evaluation was conducted using a larger and class-balanced dataset to obtain more reliable performance estimates. Specifically, the test set contained only original handwriting samples with equal representation across the four classes. For model training, LiquidGAN was used to augment the original training data, resulting in 200 images per class from each generator, including both real and synthetic samples.

#### 2.3.1. Generative Adversative Network

The feature extraction and image generation process for the generative component of the GAN consisted of five stages: (1) feature extraction, (2) Fast Dynamic Unit, (3) Liquid Neural Inertial GRU, (4) feature fusion, and (5) image generation.

#### 2.3.2. Liquid Generator

**Figure 2:**
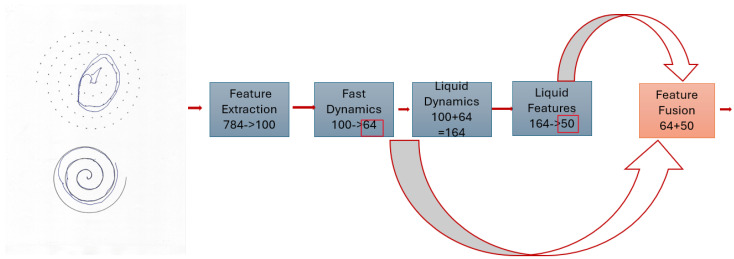
A breakdown of the dynamic learning GAN model, where the features are extracted from the liquid and the fast layers before the features are fused.

The feature extraction for the generative model was performed using liquid neurons, neural ODE integration, and physics-based dynamics. Stage 3 of the liquid generator consists of liquid Neural Inertial GRU combined as the physics-based equation (Equations (1)–(7)). Using these, liquid features were extracted from the handwriting dataset (Figure 2). The pseudocode for this dynamic learning in the GAN model is shown in Algorithm 1.

Given the state variables $\left(x,\dot{x}\right)=\left(\mathrm{position},\mathrm{velocity}\right)$, the dynamics are modeled as:

(1) $$m\ddot{x}+c\dot{x}+kx=f\left(x\right),$$

where

(2) $$\begin{array}{cc} f(x) & =\tanh(x), \end{array}$$

(3) $$\begin{array}{cc} \mathrm{spring}\;\mathrm{force} & =-|k|\;x, \end{array}$$

(4) $$\begin{array}{cc} \mathrm{damping}\;\mathrm{force} & =-|c|\;\dot{x}, \end{array}$$

(5) neuralforce=tanh(x),

(6) totalforce=springforce+dampingforce+neuralforce,

(7) $$\begin{array}{cc} \mathrm{acceleration} & =\frac{\mathrm{total}\;\mathrm{force}}{\left(\left|m\right|+10^{-6}\right)\cdot \tau \left(x\right)}, \end{array}$$

where τ(x)=0.5·σ(Wx+b)+0.1 is the adaptive time constant produced by tau_net, and σ(·) denotes the sigmoid function.
**Algorithm 1** Liquid Inertial Dynamics (ODE Right-Hand Side)**Require:** 
states = (position, velocity), m,b,k**Ensure:** 
(velocity,acceleration) 1:τ=σ(Linear(position))·0.5+0.1 2:$F_{\mathrm{spring}}=-\left|k\right|\cdot \mathrm{position}$ 3:$F_{\mathrm{damping}}=-\left|b\right|\cdot \mathrm{velocity}$ 4:$F_{\mathrm{neural}}=\tanh\left(\mathrm{position}\right)$ 5:$F_{\mathrm{total}}=F_{\mathrm{spring}}+F_{\mathrm{damping}}+F_{\mathrm{neural}}$ 6:$acceleration=F_{\mathrm{total}}\;/\;\left(|m|+\epsilon \right)\;/\;\tau$ 7:**return **(velocity,acceleration)**Require:** 
position x, velocity v, time span $\left[t_{0},t_{T}\right]$**Require:** 
Learnable parameters: mass *m*, damping *c*, spring *k***Ensure:** 
Updated position $x^{*}$ after ODE integration

The physics-based Equations (1)–(7) were used for modeling tremor dynamics, where −kx represents elastic restoring forces in muscles and tendons, $-c\dot{x}$ represents viscous resistance in muscle tissue, and tanh(x) is for neural modulation and non-linear muscle activation. These dynamic equations simulate tremor mechanics observed in EPS, where spring and damping terms correspond to muscle elasticity and viscosity, and tanh(x) represents non-linear muscle activation.

An ODE solver was used to track changes in latent features over continuous time, allowing the network to learn temporal dependencies inherent in handwriting dynamics. The neurons were used on static feature vectors based on the tremor modeling Equations (1)–(7). The liquid features were extracted from the handwriting dataset (Figure 2).

The generator of the GAN model utilized feature extraction from both liquid and fast layers. The final feature fusion (Stage 4) before the image generator was performed by combining both liquid neural ODE dynamics and the fast temporal features (Figure 2).

Dynamic features were extracted and the final features fused before classification. One hundred features were extracted from the initial features. Then, from the 100, 64 rapid temporal features were extracted. Subsequently, the 100 features were combined with fast dynamics to make a total of 164 features. Afterwards, 50 liquid features, such as viscosity and pressure-driven motion, were extracted. Finally, 64 fast dynamic features and 50 liquid features were fused together (Figure 2).

### 2.4. Image Generator

The final generator consisted of four transposed convolutional layers, each followed by a ReLU activation function. Then, three convolutional layers were applied to produce the output image. A standard convolutional neural network (CNN)-based discriminator was employed, comprising five convolutional blocks, each followed by a classification head and ReLU activation.

### 2.5. Discriminator

The discriminator uses a fully connected linear layer with sigmoid activation for final classification (Table 1). The whole model has 4 convolutional layers with an input layer and LeakyReLu activation function.

#### 2.5.1. HOG Feature Extraction

For downstream classification, static handwriting images were converted into gradient-based representations using the Histogram of Oriented Gradients (HOG) algorithm. The parameters were specified by gradually increasing the window size, block size, block stride, cell size, and number of bins until the highest performance of the model was achieved (Table 2): a window size of 200 was selected, a number of bins of 9, and block stride and cell size of 8.

#### 2.5.2. Clinical Feature Extraction

A total of 55 Clinical features were extracted from the handwriting images, and the most significant 30 features were determined using Pearson r and Spearman rho. Then, normalized and Z values were calculated to determine the features changes with severity. SHAP values were determined to measure the discriminative power, class-level importance, and the overall feature contribution, respectively. This process was performed on the total (synthetic + original) images as well as the original images only. These were correlated to the HOG features.

### 2.6. Classification

The final stage was classification of extrapyramidal symptoms (EPS) severity using HOG-extracted handwriting features. A fully connected Artificial Neural Network (ANN) was designed to classify samples into four categories: Not on Antipsychotic, EPS Mild, EPS Moderate, and EPS Severe. The network consists of an input layer, hidden layers, and an output layer. The HOG features were processed through an input layer to 256 neurons, then through 2 hidden layers with ReLU activation, and a final output layer with a softmax function for 4-class classification (Table 3). The model was trained using the Adam optimizer with a learning rate of 0.001 (Table 4).

#### 2.6.1. Network Architecture

The overall pipeline used handwriting spatial pattern recognition for the final classification.

#### 2.6.2. Training Configuration

The input data were of size 64×64 and in color, allowing capture of intensity details. A moderate batch size of 32 was selected (Table 5). A total of 50 epochs was applied to train the model. A generator learning rate of 0.0002 was leveraged for the model to learn fine details seen in handwriting of patients. A discriminator learning rate of 0.0001 was employed, slightly lower than the generator to prevent overpowering, with an Adam optimizer (Table 5).

After HOG feature extraction, an ANN was utilized for feature classification. The evaluation metrics for the final model classification (Accuracy, Precision, Recall, F1 Score, Confusion Matrix) were performed only on the test set (80 original samples, 20 participants per class). The ANN was trained on 200 samples per class (synthetic + original images).

### 2.7. Comparative Studies

**Figure 3:**
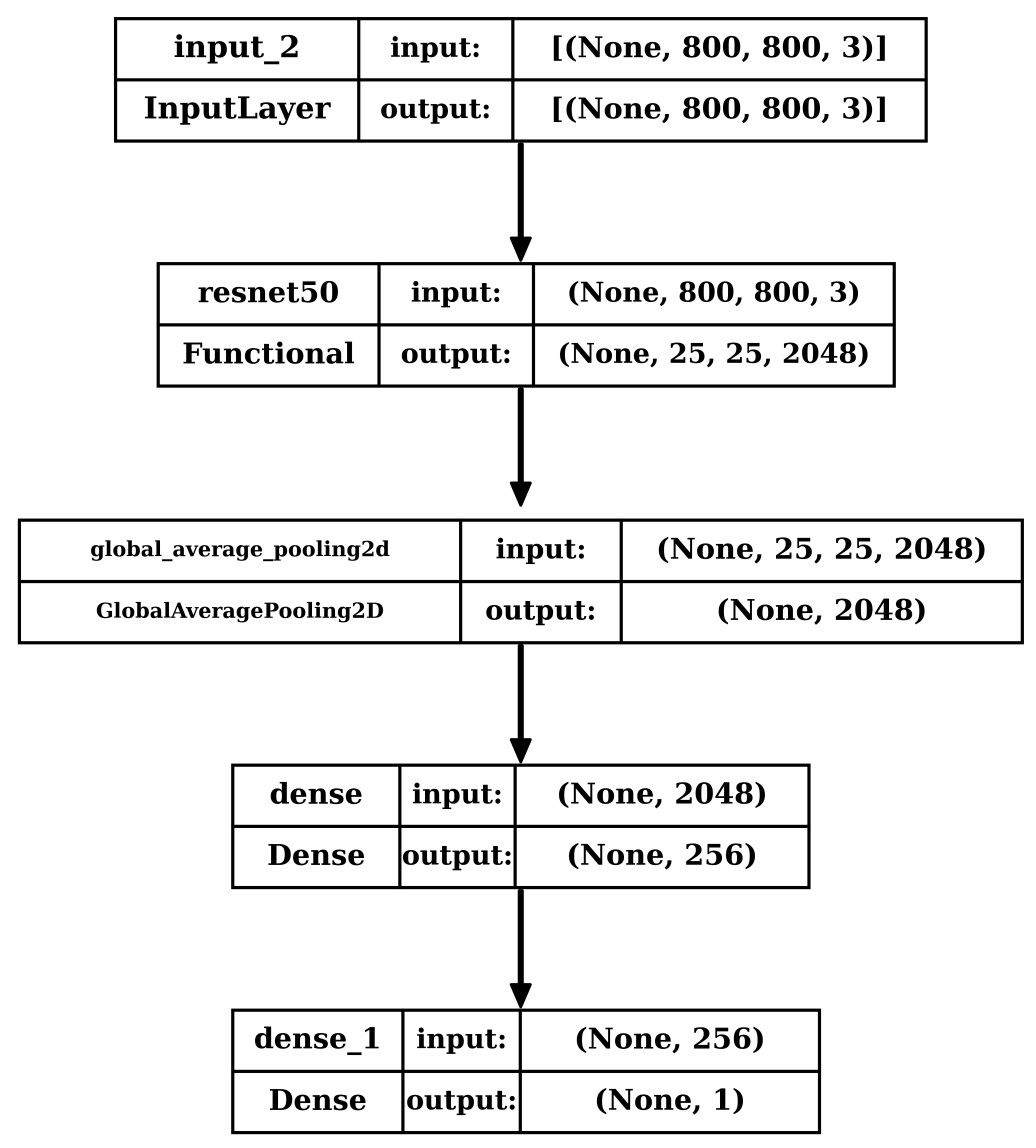
The feature extraction component of the Resnet model.

A pretrained ResNet model was employed for feature extraction and classification. All layers of the ResNet model were kept trainable except the classification head. It was used for both binary classification (Not on antipsychotics vs. EPS) and multiclass classification (Not on antipsychotics, Mild, Moderate, and Severe EPS) Figure 3. The dataset of 211 images was split into separate sets for binary and multiclass tasks.

The model was trained using an input resolution of 800 × 800 × 3 with a pretrained ResNet50 backbone. The Adam optimizer was employed, and binary cross-entropy was used as the loss function. Training was conducted for 20 epochs.

### 2.8. Experimental Design and Statistical Analysis

The experimental evaluation was designed to assess both the generative performance of the proposed Liquid GAN and the classification accuracy of the integrated HOG-ANN model.

#### 2.8.1. Comparative Design

Three generative architectures were compared:

(1) a baseline GAN,
(2) StyleGAN,
(3) Proposed Liquid GAN.

Synthetic handwriting images generated by each model were used to augment the training dataset. The downstream classification task was then performed using three classifiers: a CNN, a CNN-ANN hybrid, and the HOG-ANN architecture. This design enabled performance comparisons across both generation and classification dimensions.

#### 2.8.2. Training–Testing Protocol

The dataset was divided into training and testing sets. The test set consisted solely of original handwriting samples, with 20 samples per class (control, mild, moderate, and severe) to ensure balanced evaluation. The training set contained 118 original samples and was augmented using LiquidGAN, resulting in a balanced dataset of 200 samples per class comprising both real and synthetic images.

To assess model robustness and generalizability, stratified five-fold cross-validation and participant-level cross-validation were performed. For each fold, training was conducted using a balanced mixture of real and synthetic samples, while final performance was evaluated exclusively on the independent test set containing only original images.

#### 2.8.3. Evaluation Metrics

For the generative models, the Fréchet Inception Distance (FID), Inception Score (IS), Precision, Recall, and Kernel Inception Distance (KID) were calculated to assess realism and diversity of generated images.

For classification, Accuracy, Precision, Recall, F1-score, and a Confusion Matrix were computed on the held-out test set. Statistical significance between model performances was tested using a two-tailed paired *t*-test with α=0.05.

#### 2.8.4. Visualization and Interpretation

To illustrate class-specific handwriting variations, the average image of the Not on antipsychotic group was subtracted from class-averaged images (Mild, Moderate, Severe) to generate heat maps. Red regions denote higher edge activity relative to baseline, while blue regions represent lower activity. These visualizations support the quantitative metrics by highlighting the spatial locations most affected by extrapyramidal symptoms.

#### 2.8.5. Statistical Analysis of Clinical Features

Pearson’s correlation coefficient (*r*) and Spearman’s rank correlation coefficient (ρ) were calculated to examine both linear and monotonic relationships between the extracted handwriting features and EPS severity. For Pearson’s correlation, each handwriting feature was correlated with the EPS severity score, where severity was encoded as an ordinal scale (No EPS = 0, Mild = 1, Moderate = 2, Severe = 3). Features were subsequently ranked according to their Pearson’s *r* values to identify those most strongly associated with increasing EPS severity. Spearman’s ρ was calculated to assess monotonic relationships that may not be strictly linear. In addition, row-wise Z-scores were computed from the per-class feature means and used to generate the correlation heatmap, providing a normalized visualization of feature variations across EPS severity levels.

## 3. Results

The experiment results are presented in four parts: (a) comparison of GAN performance, (b) classification results, (c) ablation study, and (d) feature analysis.

### 3.1. Dynamic GAN Model

Table 6 shows the model performance with the original GAN, StyleGAN, and our model. It also shows our GAN model in combination with the CNN and Transformer feature extraction. The GAN performance is evaluated with the Fréchet Inception Distance, Inception Score, Precision and Recall, Likeness Score, Mode Score, and Classification Accuracy. The Accuracy, Precision, Recall, and F1 Score of the classification model with each GAN are demonstrated in Table 7.

The GAN-HOG-ANN model had the lowest FID, compared to the StyleGAN and Our Model. The Inception score was 0.04 higher than the other two models (Table 7). The KIDs were higher in the GAN-HOG-ANN model, of 0.1145, which was almost similar to the Our Model-HOG-ANN KID Score of 0.1148. There is a significant difference between GAN-HOG-ANN and StyleGAN-HOG-ANN in FID, IS, Precision, KID. However, at *p* < 0.01 there is no significant difference between our model and the original GAN model.

### 3.2. Classification Model

Generative models have the ability to produce identical or similar synthetic images, which allows training of deep learning models to be more robust. Our liquid neuron-based generative model shows the highest performance (Table 7). The HOG-ANN feature extraction and classification was compared with original GAN and StyleGAN. Our model had the highest Accuracy, Precision, Recall, and F1 Score of 70%, which is approximately 21%, 19%, 21%, and 25% higher than the HOG-ANN alone and 20%, 11%, 20% and 26% StyleGAN in Accuracy, Precision, Recall and F1 Score, respectively (Table 7). There was a significant difference between all models (*p* < 0.01).

### 3.3. Binary Classification

With the EPS patient versus the healthy control, the binary classification model at 10-fold cross-validation (Table 8) was preformed, with Accuracy, Precision, Recall, AUC values of 92%, 97%, 89%, and 96%, respectively.

When comparing the same ResNet50 model classification with Mild, Moderate, Severe, and Not on antipsychotic groups, there was significant difference in Accuracy, Precision, Recall, AUC, sensitivity and specificity with a *p* < 0.01 (Table 8).

The proposed model, when compared to the binary classification into NON EPS versus EPS groups, worked with high Accuracy, Precision, Recall and F1 Score of 97%, 97%, 97%, and 97%, respectively (Table 9).

### 3.4. Ablation Study

Convolutional neural networks (CNNs) are the most standard spatial feature extraction method, capturing edges, textures, and object parts [24]. This deep learning model was compared with HOG feature extraction and ANN for classification (Table 10). The CNN for both feature extraction and classification performed the lowest, with Accuracy, Precision, Recall, and F1 Score of 21%, 5%, 21%, and 8%, respectively.

The hybrid model employing an ANN classifier achieved the highest performance, with Accuracy, Precision, rRcall, and F1-score of 70%, 70%, 70%, and 70%, respectively. To further evaluate the model’s generalizability, participant-level cross-validation was conducted, where all samples from an individual participant were assigned exclusively to either the training or testing set. Under this more rigorous evaluation protocol, the model achieved Accuracy of 63%, Precision of 63%, Recall of 63%, and F1-score of 61%. The observed reduction in performance suggests that participant-level validation presents a more challenging and clinically realistic scenario by accounting for inter-participant variability and preventing potential information leakage between training and testing sets. Nevertheless, the results demonstrate that the proposed hybrid framework maintains reasonable predictive capability when evaluated on previously unseen participants.

These results (Table 10) further confirm that HOG features, which explicitly capture edge orientation, stroke direction, and curvature information, are more effective for spiral-based handwriting analysis than CNN-derived local features. As the Archimedean spiral is characterized by continuous directional and curvature variations, HOG descriptors are better suited to preserving these discriminative patterns associated with EPS severity, resulting in superior classification performance.

**Figure 4:**
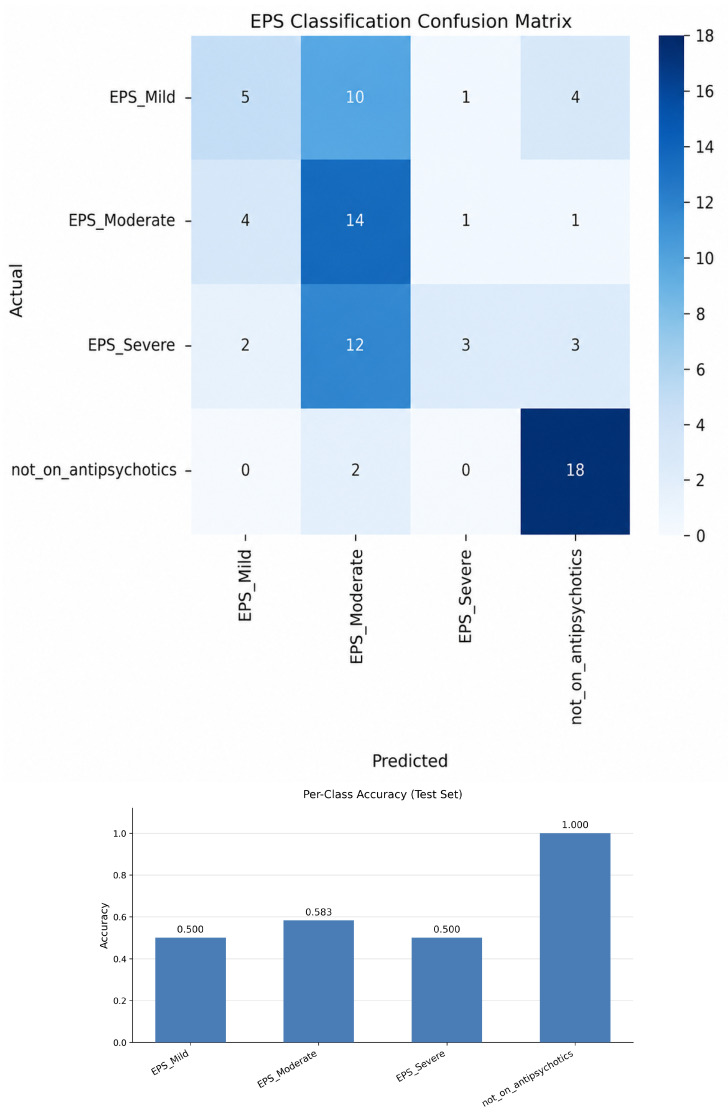
The overall confusion matrix and accuracy of each class as a histogram for the model.

The highest Classification Accuracy and Precision were from the HOG-ANN hybrid model with our GAN model (Table 10). The confusion matrix for the Liquid GAN HOG-ANN classification model showed almost equal true positive and true negative values in all four classes (Figure 4).

The EPS mild and EPS severe group had the lowest accuracy of 50% compared to the Not on antipsychotic, Moderate, and Severe EPS classes. The Not on antipsychotics group had 100%, while the Moderate group had 58%, respectively (Figure 4).

### 3.5. Feature Analysis

**Figure 5:**
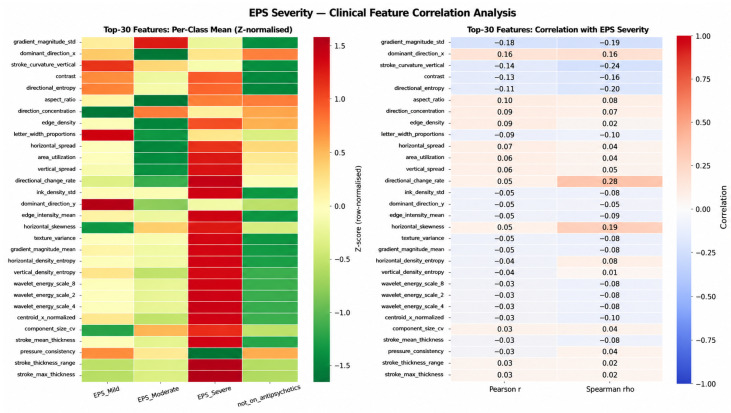
Clinical features normalized to the severity score (heat map) along with its Pearson *R* and Spearman ρ.

The HOG features extracted from handwriting patterns were used in classification into Mild, Moderate, and Severe EPS groups. Figure 5 shows the relative importance of each clinical feature in the classification process.

The Severe class showed the most variation (red) when compared with the Not on antipsychotics group (green) in the heatmap (Figure 5). Stroke thickness exhibited the greatest variation, followed by wavelet energy and directional change rate. All other severity classes demonstrated at least one to two features that varied compared with the Not on antipsychotics group. The Mild class showed the greatest changes in dominant direction and letter width, followed by stroke curvature, whereas the Moderate group demonstrated notable changes in gradient magnitude (Figure 5). The Spearman correlation showed the most significant changes among the ranked features (red), while Pearson’s r showed more neutral correlations (blue).

### 3.6. Handwriting Dominant Features

With the onset of EPS, cogwheel rigidity, bradykinesia, and tremor take place. These alter the handwriting of the patients. Our prediction model is based on features extracted from the Archimedean spiral. There were five most dominant features (Figure 5) that are used for the Not on antipsychotics, Mild, Moderate, and Severe classification in our prediction model (Table 7).

### 3.7. Location of Dominant Features

**Figure 6:**
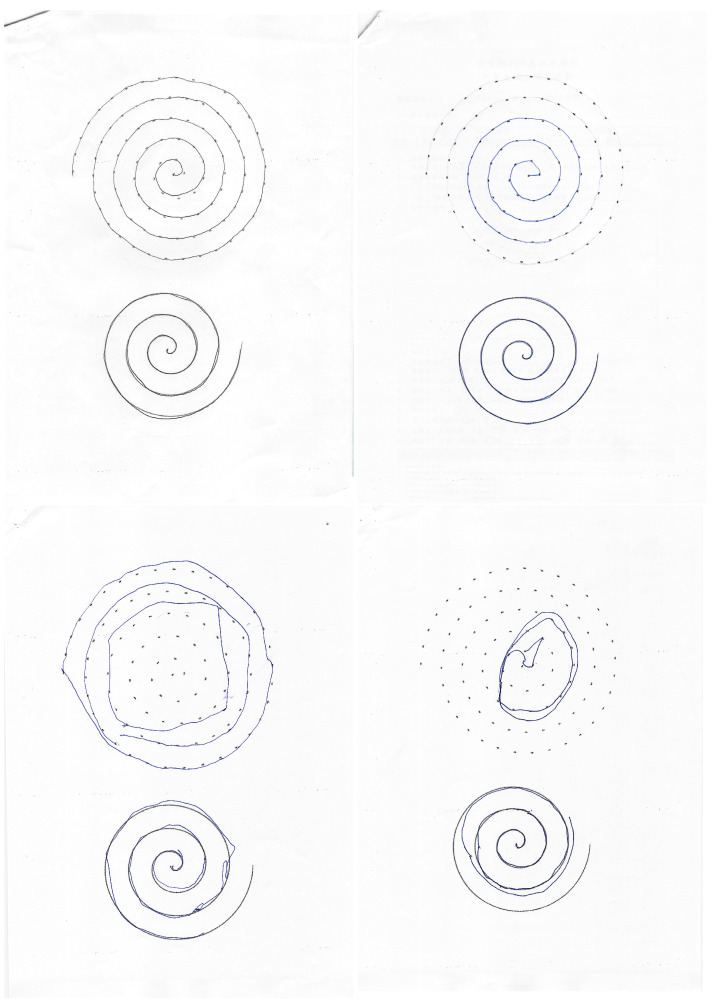
The demonstration shows how the handwriting pattern alters with the severity of the EPS.

**Figure 7:**
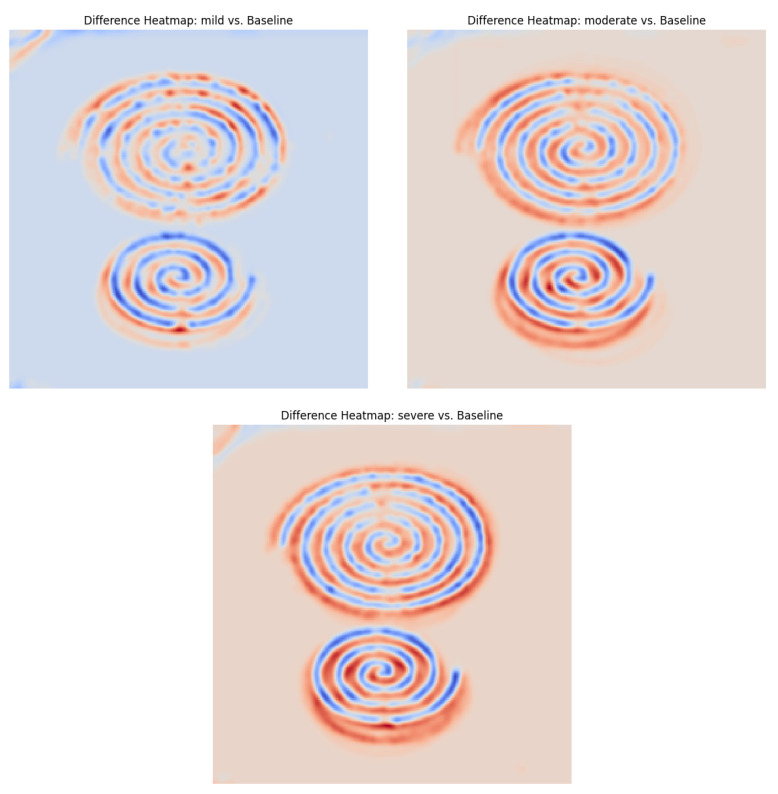
Heat map of the averaged class representation superimposed onto the other classes.

The changes in the stroke, the speed, and the thickness of the stroke all occur with the intake of AP medication (Figure 6). The location of exact feature importance also varies with the severity of EPS (Figure 7).

The Archimedean spiral from one representative image from each class shows prominent changes in the pattern of the handwriting between patients. There was a vast change in the handwriting of the Severe and Moderate classes, with patients not being able to follow the Archimedean spiral pattern. The Mild to Not on antipsychotics groups showed more ability to follow the fine circularity patterns of the Archimedean spiral (Figure 6).

The average of the base (Not on antipsychotics) group was subtracted from the average of the other three groups. Both the Severe and the Moderate heatmaps had more red area, which shows that the test group had more edge activity than the baseline. The Mild compared to the baseline had more blue area, showing less edge activity than the baseline (Figure 7).

### 3.8. Model Interpretability

**Figure 8:**
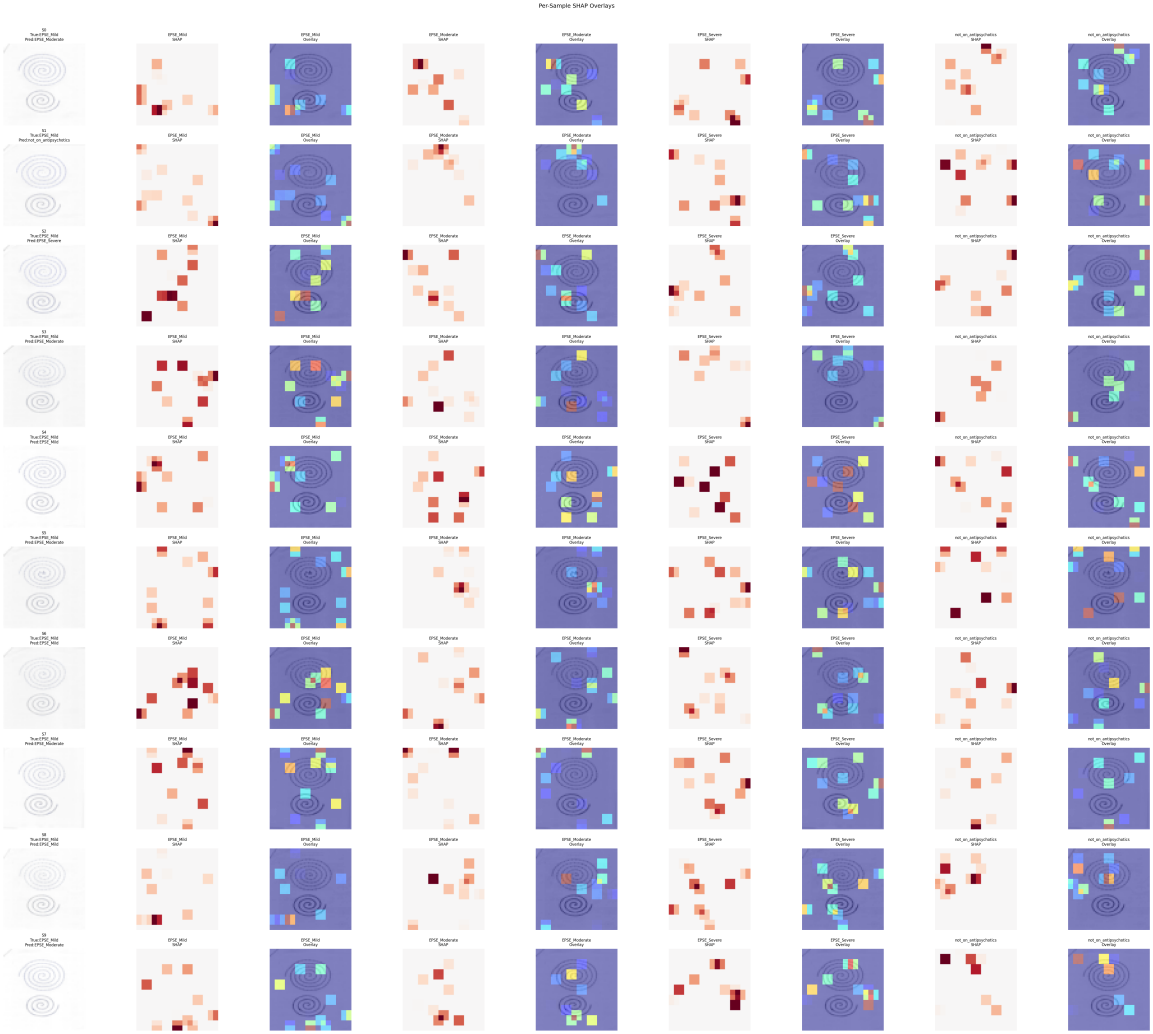
SHAP overlay into the Mild, Moderate, Severe and Not on antipsychotics classes.

Figure 8 provides a visual explanation of the regions that contribute most strongly to the model’s classification decisions. The SHAP overlay maps show that the control (Not on antipsychotics) class is characterized by predominantly blue regions, indicating areas that negatively contribute to EPS severity predictions and support classification as a healthy control. In contrast, the Mild, Moderate, and Severe EPS classes exhibit distinct red-highlighted regions, representing features that positively contribute to the corresponding severity predictions. The variation in the spatial distribution and intensity of these highlighted regions suggests that the model learns different discriminative handwriting characteristics for each severity level.

## 4. Discussion

Extrapyramidal symptoms are drug-induced side effects caused by antipsychotic medications. These side effects directly affect handwriting and motor control due to symptoms such as bradykinesia, ataxia, and cogwheel rigidity. The severity of EPS varies between individuals and depends on the specific type of medication prescribed. Currently, psychiatrists cannot reliably predict who will develop EPS or the extent of its severity. Clinical assessment typically relies on physical examinations followed by medication adjustments. Currently, there is no alternative to this trial-and-error method of AP prescription or remote assessment for EPS.

### 4.1. Interpretation of Findings

Our findings demonstrate that handwriting patterns are significantly altered with the severity of EPS, making handwriting a valuable biomarker for monitoring these side effects. Since the robustness of machine learning models depends on both the quantity and quality of training data, we employed generative models to augment the dataset. Specifically, we generated 200 synthetic handwriting images per class. To ensure no synthetic data were present in the test set, an explicit test set was utilized at each fold. To compare our model with existing models, a baseline GAN and ResNet were applied. When evaluated against StyleGAN and the original GAN, our proposed dynamic learning GAN model achieved superior performance. The model attained a Precision of 84%, representing approximately a 20% improvement over baseline models. In the context of GANs, Precision reflects the realism of generated images compared to real samples, whereas Recall captures the model’s ability to generate a diverse set of images.

The higher performance can be attributed to the model’s integration of a physics-based inertial equation and the differential equation-based liquid neurons (Equations (1)–(7)), which provide dynamic learning capabilities and improve feature generation. This could also be due to the liquid state of the neurons with adaptive time constants. EPS is associated with bradykinesia, rigidity, and tremor, all of which cause changes in fine motor skills. Handwriting patterns usually have varying stroke thickness and amplitude. Our model, with its liquid generator, has the capability to learn fine changes in handwriting patterns that a traditional neural network-based generator could not identify and replicate in its synthetic images [25].

### 4.2. Binary vs. Multiclass Classification

The proposed LiquidGAN achieved 70% Accuracy and Precision in classifying EPS severity across four categories: Control, Mild, Moderate, and Severe. To further assess the model’s generalizability, participant-level cross-validation was conducted, whereby data from each participant were assigned exclusively to either the training or testing set. Under this more stringent evaluation protocol, the model achieved 63% Accuracy and Precision. The observed reduction in performance is expected, as participant-level cross-validation better reflects real-world clinical deployment by preventing information leakage between training and testing sets and accounting for inter-participant variability. This result suggests that the classification task becomes more challenging when the model is required to generalize to previously unseen participants. Nevertheless, the participant-level results demonstrate that LiquidGAN maintains reasonable predictive performance and robustness under a clinically realistic evaluation setting.

The pretrained ResNet50 attained 92% Accuracy and 97% Precision under binary classification (Table 8); however, its performance declined substantially under multiclass classification. While pretrained models can improve classification outcomes in binary settings, multiclass classification is inherently more challenging when only a limited dataset is available. The dataset contains only 211 images and is imbalanced across classes, which may limit the effectiveness of pretrained features in capturing subtle inter-class differences [26]. Incorporating an additional generative model may help mitigate this limitation. Notably, the proposed LiquidGAN achieved 97% accuracy for binary classification of EPS versus non-EPS cases.

### 4.3. GAN Evaluation

Evaluation using Fréchet Inception Distance (FID) showed that GAN-HOG-ANN model achieved a score of 141.03, substantially lower than both StyleGAN and the liquid GAN, indicating closer alignment between synthetic and real image feature distributions [20]. Moreover, the Kernel Inception Distance (KID) score was reduced to 0.1145. Though the GAN-HOG-ANN model had a better GAN evaluation compared to the StyleGAN and our model. There was no significant difference (*p* < 0.01) between our model and the traditional GAN model (Table 6). In contrast, our model performed with higher Accuracy, Precision, Recall, and F1 Score with respect to the comparative models (Table 10). This might be attributed to the fusion between the liquid and fast features, which captures both temporal and spatial features. Our Liquid GAN model, with built-in liquid time constants, can detect temporal irregularities from spatial artifacts [27].

Handwriting patterns do change with the intake of antipsychotic medications due to the tremor and rigidity resulting from EPS. Our classification model suggests this with an EPS predicting Accuracy and Precision of 70%. The classification is achieved using HOG features extracted and ANN classification of these features. The classification model utilizing CNN for feature extraction and classification achieved Accuracy and Precision of 21% and 5%, respectively. With the addition of ANN classification, the model improved to Accuracy of 43% and 36%, respectively. Even with a Deep Neural Network (DNN) for feature extraction and ANN for classification, Accuracy and Precision were only 35% and 57% respectively, proving that HOG had the highest performance [28]. This is due to the fact that HOG is highly sensitive to edge detection directionality and curvature, while CNNs are for learning spatial features, and they often learn local textures and edge patterns. EPS handwriting changes, such as micrographia and tremor-induced irregularities, are more global distributions. Also, CNN models require large datasets to train, while HOG focuses on global distribution and can detect small irregular curvature in spirals and loops.

The liquid neural network-based GAN model learns both the temporal and spatial patterns of the Archimedean spiral. The temporal aspects of handwriting include stroke velocity and sequence, timing, rhythm, and dynamic pressure. Spatial aspects of handwriting include the character shape, stroke thickness, character spacing, and style consistency. EPS is associated with changes in stroke timing and rhythm. The character size and spacing also showed changes with EPS severity. These are further verified by the feature patterns and handwriting heatmaps.

### 4.4. Clinical and Technical Implications

Our study demonstrated several handwriting feature changes associated with increasing EPS severity, particularly directional change rate, stroke thickness, wavelet energy, texture variance, and edge intensity. These features exhibited the greatest variation in the severe EPS class, whereas relatively fewer feature changes were observed in the mild and moderate classes. Stroke curvature and letter width proportion were among the most important features distinguishing the mild EPS group (Figure 5). Figure 5 also highlights inconsistencies in the raw Archimedean spiral patterns of the moderate and severe groups, with greater edge activity (red regions) observed when comparing the Not on antipsychotics group with the Mild and Moderate EPS groups.

Letter width proportion was another important feature for classification, reflecting irregular letter formation and inconsistent spacing. These abnormalities may be associated with the onset of tremor. Furthermore, discontinuities in the Archimedean spiral patterns are evident in Figure 6. Such disruptions in continuous writing are consistent with the presence of tremor, which has been reported at frequencies around 5 Hz in patients experiencing EPS [16]. Notably, the most influential regions identified during feature extraction and classification (red regions) varied across severity levels and were predominantly located toward the outer regions of the spirals (Figure 8). This suggests that the model distinguished between Mild, Moderate, and Severe EPS classes based, in part, on the spatial distribution of handwriting abnormalities.

Stroke curvature differed between the Not on antipsychotics group and the Mild EPS group but was less prominent in the Moderate and Severe classes. Patients with EPS commonly exhibit micrographia and altered letter formation due to parkinsonian symptoms associated with antipsychotic treatment [25]. Variations in cursive writing have also been associated with cognitive function [29]. Similar patterns can be observed in Figure 6 and Figure 7, where the smaller Archimedean spirals in the lower sections display increased circularity and looping characteristics.

Stroke direction and stroke thickness were also important features associated with EPS severity, particularly in the Severe class (Figure 5). These changes are visually apparent in Figure 6, where the Moderate and Severe groups exhibit thicker strokes and greater texture variation. Such alterations may reflect rigidity and dystonia in patients with EPS, resulting in fluctuations in writing pressure and motor control.

The Spearman and Pearson correlation analyses demonstrated that the most influential features associated with EPS severity were directional change rate, horizontal skewness, and dominant direction (Figure 5). Alterations in writing direction and the rate of directional changes may reflect the effects of bradykinesia and rigidity [2,3], which are commonly observed in patients receiving antipsychotic medications. Furthermore, the identification of horizontal skewness as a significant feature suggests the possibility of directional changes in the plane of handwriting movements, potentially due to dystonia-induced sudden jerky motions [30]. Notably, the stronger correlations observed in the Spearman analysis suggest that changes in these handwriting features are not linearly proportional to EPS severity. This finding indicates a nonlinear relationship between feature variation and symptom progression, whereby feature changes may occur at different rates across levels of severity.

### 4.5. Limitations

One limitation of this research is the dataset. While there is a diverse age and gender group, there is, however, a geographical and linguistic limitation. Although our model achieves an Accuracy of 70%, the weakness of this study is the variable type, dosage, and duration of medication. Furthermore, clinical diagnoses are seldom based on a single modality; incorporating multiple modalities affected by symptoms like dystonia, cogwheel rigidity, and bradykinesia could provide a more comprehensive assessment of disease progression. To facilitate this as part of our future research, we can develop a multi-modal model combining handwriting and voice modalities. This would enable healthcare professionals to make more informed and personalized diagnostic decisions.

## 5. Conclusions

This study demonstrated that handwriting patterns have the potential of being used as a non-invasive tool for detecting and monitoring EPS in patients receiving antipsychotic medication. By integrating a Liquid Generative Adversarial Network (Liquid-GAN) with Histogram of Oriented Gradients (HOG) feature extraction, we developed a dynamic model capable of learning both spatial and temporal handwriting characteristics.

The proposed model achieved 70% accuracy and precision in classifying four EPS severity levels, outperforming traditional GANs, StyleGAN, and ResNet50 as baselines. The liquid neuron architecture introduced dynamic learning through differential equations, enabling the model to capture tremor- and rigidity-related oscillations in handwriting. These findings support the clinical validity of handwriting-based digital biomarkers and highlight the potential of physics-informed generative models for medical diagnostics when training data are limited.

Future work will focus on developing a multimodal framework that combines handwriting analysis with additional clinical and behavioral biomarkers. Such an approach is expected to improve robustness, generalizability, and clinical applicability in real-world deployment scenarios.

## Figures and Tables

**Table 1:** Liquid GAN Discriminator Architecture for Handwriting Image Classification.

Layer	Type	Input	Output	Activation
Input Layer	Conv2d	3×64×64	32×32×32	LeakyReLU(0.2)
Conv Layer 1	Conv2d	32×32×32	62×16×16	LeakyReLU(0.2)
Conv Layer 2	Conv2d	62×16×16	64×8×8	LeakyReLU(0.2)
Conv Layer 3	Conv2d	64×8×8	128×4×4	LeakyReLU(0.2)
Conv Layer 4	Conv2d	128×4×4	256×2×2	LeakyReLU(0.2)
Pooling	AvgPool2d	256×2×2	256×1×1	None
Flatten	Flatten	256×1×1	256	None
Output Layer	Dense	256	1	Sigmoid

**Table 2:** HOG Feature Extraction Parameters.

Parameter	Value
Window Size (winsize)	200
Block Size (blocksize)	16
Block Stride (blockstride)	8
Cell Size (cellsize)	8
Number of Bins	9

**Table 3:** ANN Architecture for HOG Features from Handwriting Data to Determine EPS Classification.

Layer	Type	Input	Output	Activation
Input Layer	Dense	1764	256	None
Hidden Layer 1	Dense	256	128	ReLU
Hidden Layer 2	Dense	128	64	ReLU
Output Layer	Dense	64	4	Softmax

**Table 4:** ANN Hyperparameters.

Parameter	Value
Input Features	1764 (HOG Features)
Hidden Layers	3 (256 → 128 → 64)
Activation Function	ReLU
Optimizer	Adam
Learning Rate	0.001 (default)

**Table 5:** GAN Model Training and Data Parameters.

Parameter	Value
Input Data	
Image Size	64×64
Color Channels	3 (RGB)
Batch Size	32
Training Parameter	
Epochs	50
Generator Learning Rate	0.0002
Discriminator Learning Rate	0.0001
Optimizer	Adam

**Table 6:** Performance Comparison of GAN Variants with HOG-ANN Classifier.

Model	FID	IS	IS (Std)	Precision	Recall	KID	*p*-Value
StyleGAN-HOG-ANN	226.12	1.03	0.0109	1.00	0.06	0.1204	0.001
GAN-HOG-ANN	141.03	1.07	0.0009	1.00	0.17	0.1145	0.02
Our Model-HOG-ANN	152.81	1.03	0.0065	1.00	0.05	0.1148	0.041

**Table 7:** Comparison of Models on Accuracy, Precision, Recall, F1 Score, and Statistical Significance.

Model	Accuracy	Precision	Recall	F1	*p*-Value
HOG-ANN only	49%	51%	49%	45.0%	0.0003
GAN-HOG-ANN	50%	46%	50%	45.6%	0.0002
StyleGAN-HOG-ANN	50%	59%	50%	44.0%	0.0004
Our Model-HOG-ANN	70%	70%	70%	70.0%	0.001

**Table 8:** Comparison of Binary and Multiclass Classification Metrics for Resnet50.

Metric (%)	Acc (%)	Prec (%)	Rec (%)	AUC (%)
Multiclass Class.	57	49	57	74
Binary Class.	92	97	89	96
*T*-Test (*p*-val)	$1.08\times 10^{-6}$	$1.25\times 10^{-7}$	$1.21\times 10^{-5}$	$2.48\times 10^{-4}$

**Table 9:** Proposed Model for Binary Classification.

Metric (%)	Value
Accuracy	97
Precision	98
Recall	97
F1-Score	97

**Table 10:** Ablation Study for Proposed Model: Accuracy (%), Precision (%), Recall (%), and F1 Score (%).

Model	Acc. (%)	Prec. (%)	Rec.(%)	F1 (%)
Our Model-CNN	21	5	21	8
Our Model-CNN-ANN	43	36	43	31
Our Model-DNN-ANN	36	57	36	21
HOG-ANN only	49	51	49	45
**Our Model-HOG-ANN(Participant Lvl CV)**	**63**	**63**	**63**	**61**
**Our Model-HOG-ANN(Participant Lvl Split)**	**70**	**70**	**70**	**70**

## Data Availability

All data are be available on request.

## References

[1] McCutcheon R.A., Harrison P.J., Howes O.D., McGuire P.K., Taylor D.M., Pillinger T. (2023). Data-Driven Taxonomy for Antipsychotic Medication: A New Classification System. Biol. Psychiatry.

[2] D’Souza R.S., Aslam S.P., Hooten H.W. (2024). Extrapyramidal Side Effects. StatPearls.

[3] Mammen J.R., Adams J.L., Mangrum R., Xiao Y., Barbosa W., Tyo M., Redmond C., Carter C., Cifelli K., Cifelli R. (2025). Systematic review and consensus conceptual model of meaningful symptoms and functional impacts in early Parkinson’s Disease. npj Park. Dis..

[4] Toodayan N. (2018). James Parkinson’s Essay on the shaking palsy, 1817–2017. Med. J. Aust..

[5] Waddell L., Taylor M. (2008). A new self-rating scale for detecting atypical or second-generation antipsychotic side effects. J. Psychopharmacol..

[6] Kim J.H., Jung H.Y., Kang U.G., Jeong S.H., Ahn Y.M., Byun H.J., Ha K.S., Kim Y.S. (2002). Metric characteristics of the drug-induced extrapyramidal symptoms scale (DIEPSS): A practical combined rating scale for drug-induced movement disorders. Mov. Disord..

[7] Crespo Y., Ibañez A., Soriano M.F., Iglesias S., Aznarte J.I. (2019). Handwriting movements for assessment of motor symptoms in schizophrenia spectrum disorders and bipolar disorder. PLoS ONE.

[8] Alty J., Cosgrove J., Thorpe D., Kempster P. (2017). How to use pen and paper tasks to aid tremor diagnosis in the clinic. Pract. Neurol..

[9] Walton J. (1997). Handwriting changes due to aging and Parkinson’s syndrome. Forensic Sci. Int..

[10] Saetee P., Rueangrong T., Chanwimalueang T., Jampa-ngern S., Methawasin K., Tantisatirapong S. (2024). Enhanced Diagnosis of Parkinson’s Disease Using Digitized Archimedean Spiral Handwriting Analysis. Proceedings of the Biomedical Engineering International Conference.

[11] Wang S., Schwirtlich T., McLaughlin D., Beestrum M., Heinemann A.W. (2025). Clinical Applications and Measurement Properties of the Digitized Archimedes Spiral Drawing Test: A Scoping Review. Mov. Disord. Clin. Pract..

[12] Huang Y., Chaturvedi K., Nayan A.A., Hesamian M.H., Braytee A., Prasad M. (2024). Early Parkinson’s Disease Diagnosis through Hand-Drawn Spiral and Wave Analysis Using Deep Learning Techniques. Information.

[13] Zhu Z., Wu E., Leng P., Sun J., Ma M., Pan Z. (2025). Finger drawing on smartphone screens enables early Parkinson’s disease detection through hybrid 1D-CNN and BiGRU deep learning architecture. PLoS ONE.

[14] Hallett M. (2008). Overview of Human Tremor Physiology. Mov. Disord..

[15] Kumar P., Das R., Bhattacharjee S., Saha A., Manna H., De I., Laha M., Banerjee I. (2026). Cure-Net: A Deep Learning Framework for the Proactive Detection of Parkinson’s Disease via Automated Handwriting Analysis. SN Comput. Sci..

[16] Wysokiński A., Zwierzchowska-Kieszek A. (2024). EDEPS (Early Detection of ExtraPyramidal Symptoms): Supervised machine learning models to detect antipsychotics-induced extrapyramidal hand tremor from a mobile device built-in sensors. medRxiv.

[17] Goodfellow I., Pouget-Abadie J., Mirza M., Xu B., Warde-Farley D., Ozair S., Courville A., Bengio Y. (2020). Generative adversarial networks. Commun. ACM.

[18] Karras T., Aittala M., Laine S., Härkönen E., Hellsten J., Lehtinen J., Aila T., Ranzato M., Beygelzimer A., Dauphin Y., Liang P., Vaughan J.W. (2021). Alias-Free Generative Adversarial Networks. Proceedings of the Advances in Neural Information Processing Systems.

[19] Salimans T., Goodfellow I., Zaremba W., Cheung V., Radford A., Chen X. (2016). Improved Techniques for Training GANs. arXiv.

[20] Heusel M., Ramsauer H., Unterthiner T., Nessler B., Hochreiter S. (2017). GANs Trained by a Two Time-Scale Update Rule Converge to a Local Nash Equilibrium. Adv. Neural Inf. Process. Syst..

[21] Kumar K., Verma A., Gupta N., Yadav A. (2023). Liquid Neural Networks: A Novel Approach to Dynamic Information Processing. Proceedings of the 2023 International Conference on Advances in Computation, Communication and Information Technology (ICAICCIT).

[22] Bansal K., Kumar R. (2013). K-Algorithm A Modified Technique for Noise Removal in Handwritten Documents. arXiv.

[23] Bouwmans T., Javed S., Sultana M., Jung S.K. (2019). Deep neural network concepts for background subtraction:A systematic review and comparative evaluation. Neural Netw..

[24] Ozaydin U., Georgiou T., Lew M. (2019). A Comparison of CNN and Classic Features for Image Retrieval. Proceedings of the International Workshop on Content-Based Multimedia Indexing.

[25] Caligiuri M.P., Teulings H.L., Dean C.E., Niculescu A.B., Lohr J.B. (2010). Handwriting movement kinematics for quantifying extrapyramidal side effects in patients treated with atypical antipsychotics. Psychiatry Res..

[26] Ngiam J., Peng D., Vasudevan V., Kornblith S., Le Q.V., Pang R. (2018). Domain Adaptive Transfer Learning with Specialist Models. arXiv.

[27] Hinke-Navarro A., Nieto-Hidalgo M., Espin J.M., Tapia J.E. (2025). Enhanced Deep Learning DeepFake Detection Integrating Handcrafted Features.

[28] Dalal N., Triggs B. (2005). Histograms of oriented gradients for human detection. Proceedings of the 2005 IEEE Computer Society Conference on Computer Vision and Pattern Recognition (CVPR’05).

[29] Meulenbroek R.G.J., Van Galen G.P. (1990). Perceptual-Motor Complexity of Printed and Cursive Letters. J. Exp. Educ..

[30] Cataniag P., Suratos C.T., Diesta C.C., Ong J.N. (2025). Dystonic tremor in Writer’s cramp Mimicking primary handwriting tremor. Clin. Park. Relat. Disord..

